# Enhancing performance in numerical magnitude processing and mental arithmetic using transcranial Direct Current Stimulation (tDCS)

**DOI:** 10.3389/fnhum.2013.00244

**Published:** 2013-06-06

**Authors:** Tobias U. Hauser, Stephanie Rotzer, Roland H. Grabner, Susan Mérillat, Lutz Jäncke

**Affiliations:** ^1^Division Neuropsychology, Institute of Psychology, University of ZurichZurich, Switzerland; ^2^University Clinics for Child and Adolescent Psychiatry, University of ZurichZurich, Switzerland; ^3^Neuroscience Center Zurich, University of Zurich and ETH ZurichZurich, Switzerland; ^4^Georg-Elias-Müller-Institute of Psychology, Georg-August-Universität GöttingenGöttingen, Germany; ^5^International Normal Aging and Plasticity Imaging Center, University of ZurichZurich, Switzerland

**Keywords:** mental arithmetic, numerical magnitude processing, transcranial Direct Current Stimulation (tDCS), subtraction, number comparison, mathematics

## Abstract

The ability to accurately process numerical magnitudes and solve mental arithmetic is of highest importance for schooling and professional career. Although impairments in these domains in disorders such as developmental dyscalculia (DD) are highly detrimental, remediation is still sparse. In recent years, transcranial brain stimulation methods such as transcranial Direct Current Stimulation (tDCS) have been suggested as a treatment for various neurologic and neuropsychiatric disorders. The posterior parietal cortex (PPC) is known to be crucially involved in numerical magnitude processing and mental arithmetic. In this study, we evaluated whether tDCS has a beneficial effect on numerical magnitude processing and mental arithmetic. Due to the unclear lateralization, we stimulated the left, right as well as both hemispheres simultaneously in two experiments. We found that left anodal tDCS significantly enhanced performance in a number comparison and a subtraction task, while bilateral and right anodal tDCS did not induce any improvements compared to sham. Our findings demonstrate that the left PPC is causally involved in numerical magnitude processing and mental arithmetic. Furthermore, we show that these cognitive functions can be enhanced by means of tDCS. These findings encourage to further investigate the beneficial effect of tDCS in the domain of mathematics in healthy and impaired humans.

## Introduction

The relation between mathematical functions and the brain has fascinated neuroscientists since centuries. As one of the first modern neuroscientists, Rudolph Wagner studied the brain of the mathematical genius Carl Friedrich Gauss in the nineteenth century (Hagner, 2004). Over the last decades, a growing body of neuroimaging research led to a steadily increasing understanding of mathematical functions in the brain. The main focus of this research was on basic number processing, in particular on how numerical magnitudes are represented and processed in the brain. These questions were mainly addressed by administering number comparison tasks in which participants are presented with two numerical magnitudes and have to decide which of the two magnitudes is larger. Already Moyer and Landauer (1967) employed this task and found that reaction times and error rates were inversely related to the numerical distance between the two magnitudes. For instance, discriminating between the numbers 8 and 9 takes longer and is more error-prone than discriminating between 2 and 7. This distance effect has been replicated several times and is assumed to derive from the nature of the internal magnitude representation, which is often modeled as activation distributions on an oriented mental number line (Nieder, 2005; Nieder and Dehaene, 2009). Importantly, functional magnetic resonance imaging (fMRI) studies have observed negative correlations between numerical distance and the activation of the intraparietal sulcus (IPS), a brain region in the posterior parietal cortex (PPC; e.g., Pinel et al., 2001; Fias et al., 2003). Since this brain region has also been found to be systematically involved in other tasks that require the processing of numerical magnitudes (e.g., approximation), there is now wide consensus that the IPS holds an amodal semantic representation of numerical magnitudes (Dehaene et al., 2003; Ansari, 2007, 2008). In most neuroimaging studies, the PPC of both hemispheres is equally activated in numerical magnitude processing (Arsalidou and Taylor, 2011). Studies with split-brain patients confirm these studies by showing that both hemispheres are able to process numerosities independently (Seymour et al., 1994).

Building on the findings from basic number processing research, there is now increasing interest into more complex mathematical functions, such as mental arithmetic. Mental arithmetic contains operations which are usually taught in school, such as addition, subtraction, multiplication, or division. Solving arithmetic problems is typically accompanied by activation in a widespread network of brain regions. The composition of these regions seems to depend on the operation, problem size and the applied problem solving strategy (Grabner et al., 2009). Simple one-digit multiplications, for example, are most often solved by retrieving the solution from memory (fact retrieval), which strongly relies on the angular gyrus. Complex subtraction problems, in contrast, require procedural strategies (e.g., decomposing the problem and calculation) which strongly draw on PPC regions including the IPS. In addition, the lateralization of PPC activity also seems to depend on these factors. In a meta-analysis by Arsalidou and Taylor (2011), the authors found that in addition and partially also in subtraction tasks, the left hemisphere was more strongly involved, while multiplications were processed primarily in the right hemisphere.

One main disadvantage of human neuroimaging methods, such as fMRI, is their correlative nature. These methods indeed allow describing cortical regions which are activated during certain cognitive tasks, the causative role of these regions, however, remains unknown. Therefore, several studies investigated the effect of transcranial magnetic stimulation (TMS)—which allows to transiently inhibit cortical areas—on mathematical processing [for a review, cf. Sandrini and Rusconi (2009)]. Most TMS studies investigated the effect of PPC-inhibition on numerical magnitude tasks. The majority of these studies have reported an impairment in number comparison performance after left-sided inhibition (Göbel et al., 2001; Sandrini et al., 2004; Andres et al., 2005; Rusconi et al., 2005; Knops et al., 2006; Cappelletti et al., 2007), whereas the effect of a right-sided disruption is less consistent (Göbel et al., 2001; Sandrini et al., 2004; Andres et al., 2005; Cappelletti et al., 2007; Cohen Kadosh et al., 2007). To our knowledge, only two TMS studies investigated mental arithmetic processing. Göbel et al. (2006) inhibited the PPC during a complex double-digit addition task and were able to induce transient impairments after left-, but not after right-sided inhibition. A recent study by Andres et al. (2011) compared performance in subtraction and multiplication tasks and found increased latencies for both arithmetic operations after inhibition of the IPS in both hemispheres.

TMS is not only used to determine the causative role of cortical areas in various cognitive domains, but is also applied as a treatment in a wide variety of neurologic and neuropsychiatric disorders (Croarkin et al., 2011; Holtzheimer et al., 2012; Peng et al., 2012). However, due to the high costs of TMS and because of safety risks when enhancing cortical regions (Hummel and Cohen, 2006; Cohen Kadosh et al., 2012), a related method, namely transcranial Direct Current Stimulation (tDCS) has been increasingly used in recent years both in basic research and for treatment. TDCS modulates neural activity of cortical regions underneath the areas to which the electrodes are attached (Im et al., 2008). While anodal tDCS enhances the neural firing rate of the cortex under the anode, cathodal tDCS lowers the spiking rates beneath the cathode (Bindman et al., 1964). TDCS has been shown to be effective in modulating a variety of cognitive functions, such as working memory, pitch memory, verbal performance, or even driving behavior (e.g., Fregni et al., 2005; Vines et al., 2006b; Beeli et al., 2008a,b; Cerruti and Schlaug, 2008). Moreover, it has been shown to be successful as a remediation program for several neurologic and neuropsychiatric disorders (e.g., Boggio et al., 2008; Monti et al., 2008; Schlaug et al., 2008; Nitsche et al., 2009; Lindenberg et al., 2010).

An interesting novel application field of tDCS could be the treatment of individuals suffering from mathematical learning disorders such as developmental dyscalculia (DD). With a prevalence of 5% (Cohen Kadosh and Walsh, 2007), DD is a frequent developmental disorder with impairments in magnitude processing (Landerl et al., 2004) and mental arithmetic (Geary, 1993, 2010). If anodal tDCS could improve these impaired mathematical functions similar to the aforementioned (domain-general) cognitive processes, it undoubtedly has a large potential for the development of remediation programs for DD (Cohen Kadosh et al., 2012). Therefore, there is an urgent need for studies investigating effects of tDCS on mathematical functions.

So far, however, only one study investigated the potential beneficial effect of anodal tDCS in the domain of mathematics. Cohen Kadosh et al. (2010) investigated whether tDCS can support the acquisition of new number symbols. The participants performed a number comparison task with new number symbols while they were stimulated. The authors found that learning was enhanced applying right-sided, but not left-sided stimulation. However, due to the small sample size used in this study, the generalizability of the obtained finding can be questioned.

The aim of the present study was to evaluate beneficial effects of tDCS on both mathematical functions that are essential in the development of mathematical competence and which are also frequently impaired in DD: numerical magnitude processing and mental arithmetic. We sought to evaluate whether tDCS can enhance performance in these domains under the consideration of the unclear hemispheric lateralization. Similar to previous neuroimaging and TMS studies, we administered a number comparison task to assess effects on magnitude processing. For investigating mental arithmetic, we required participants to solve double-digit subtraction problems which strongly rely on procedural strategies. Both tasks were administered before and after different tDCS protocols in two experiments. In the first experiment, we applied anodal tDCS over the left PPC (LA) as well as over the bilateral PPC (BA). This procedure was chosen as the lateralization of the assessed tasks is not completely clear based on existing evidence. On the one hand, neuroimaging studies suggest a bilateral involvement of the PPC in magnitude processing with a lateralization trend toward the left hemisphere for subtraction problems (Arsalidou and Taylor, 2011). On the other hand, the majority of TMS studies reported stronger effects for left-sided stimulation in both tasks. To additionally test whether inhibitory tDCS has similar effects as TMS, we also assessed the effect of bilateral cathodal stimulation (BC). The second experiment was motivated by the recent study of Cohen Kadosh et al. (2010) who reported beneficial effects of tDCS over the right PPC. Consequently, we additionally tested the effect of anodal stimulation of the right PPC (RA) in both tasks. In both experiments, we applied a within-subjects design and compared the stimulation protocols with a sham (placebo) condition (S).

For the numerical magnitude processing task, we hypothesized that BA stimulation leads to performance improvements, whereas BC stimulation results in a performance decrease. We assumed that one-sided anodal stimulations (LA, RA) would result in an intermediate effect, between BA and S. For the complex subtraction task, we hypothesized that LA as well as BA would lead to improvements, because subtraction has been associated with left-hemispheric or bilateral activation. Furthermore, we speculated that BC inhibition might lead to a performance decrease.

## Materials and methods

### Experiment 1

#### Participants

In Experiment 1, the sample consisted of 21 right-handed participants (11 females) with a mean age of 22.8 years (±3.1). The subjects were recruited from the local universities and did neither have any history of neurologic or psychiatric disorders nor of DD or other mathematical disabilities. One participant had to be excluded prior to analysis due to deviant behavior during stimulation (making phone calls instead of reading silently). The local ethic committee (ethic committee of the canton of Zurich, Switzerland) approved the study, and each participant gave written informed consent.

#### Experimental design

**Figure 1:**
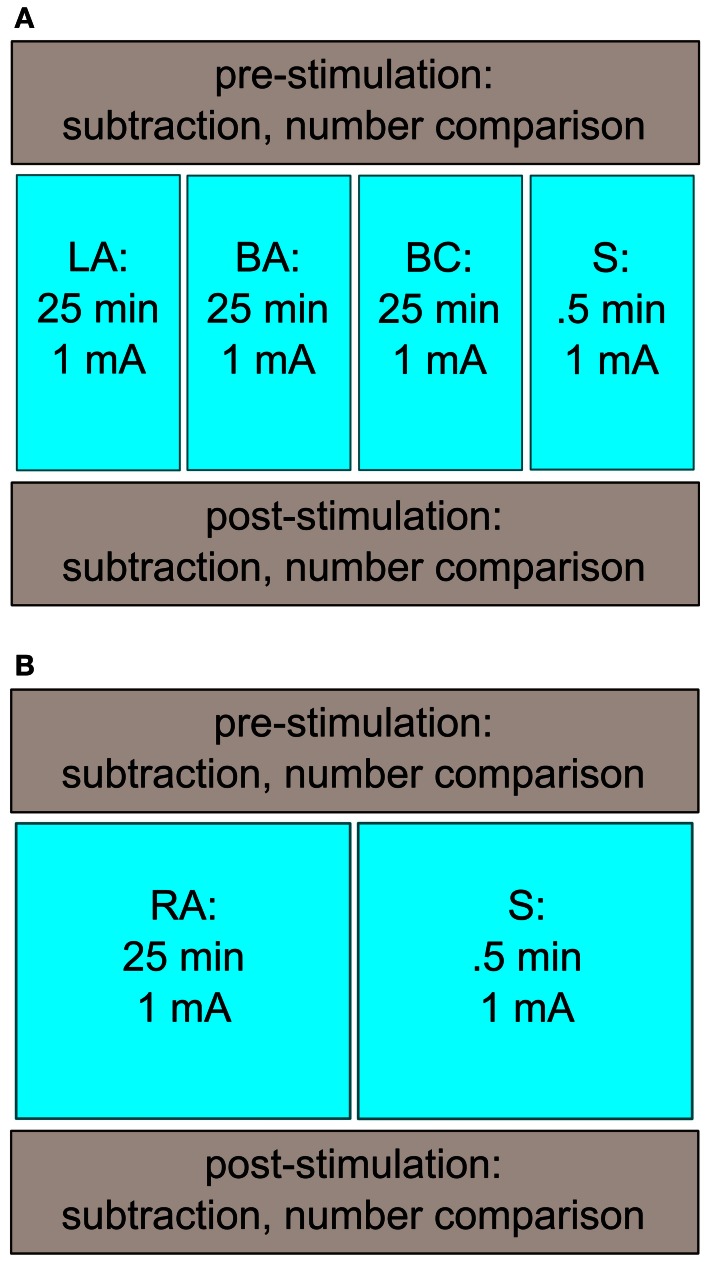
**Study design of Experiments 1 and 2. (A)** Subjects came in for four sessions. Before and after stimulation, the participants performed a number comparison and a subtraction task. The stimulation conditions (LA, BA, BC, S) were counter-balanced across sessions. **(B)** A similar study design as in Experiment 1 was used in the second experiment. The participants had to attend twice, where they received either RA or S stimulation.

The subjects participated in four sessions with a minimum intersession interval of 24 h (Figure 1A; time between two sessions: 5.3 d ± 5.0). Each session took approximately 1 h. All sessions followed the same procedure: before and after the tDCS stimulation, subjects had to solve the computerized tasks (number comparison, subtraction), presented on a 17″ computer screen using Presentation (13.0, Neurobehavioral Systems, Albany, USA). In between, tDCS was applied. A different stimulation protocol was used for each session. We applied left-sided anodal tDCS (LA), bilateral anodal tDCS (BA), bilateral cathodal tDCS (BC), and sham stimulation (S). All stimulation conditions were counter-balanced across sessions (for stimulation details, see below), and the subjects were randomly assigned to the different orders of the stimulation conditions.

**Figure 2:**
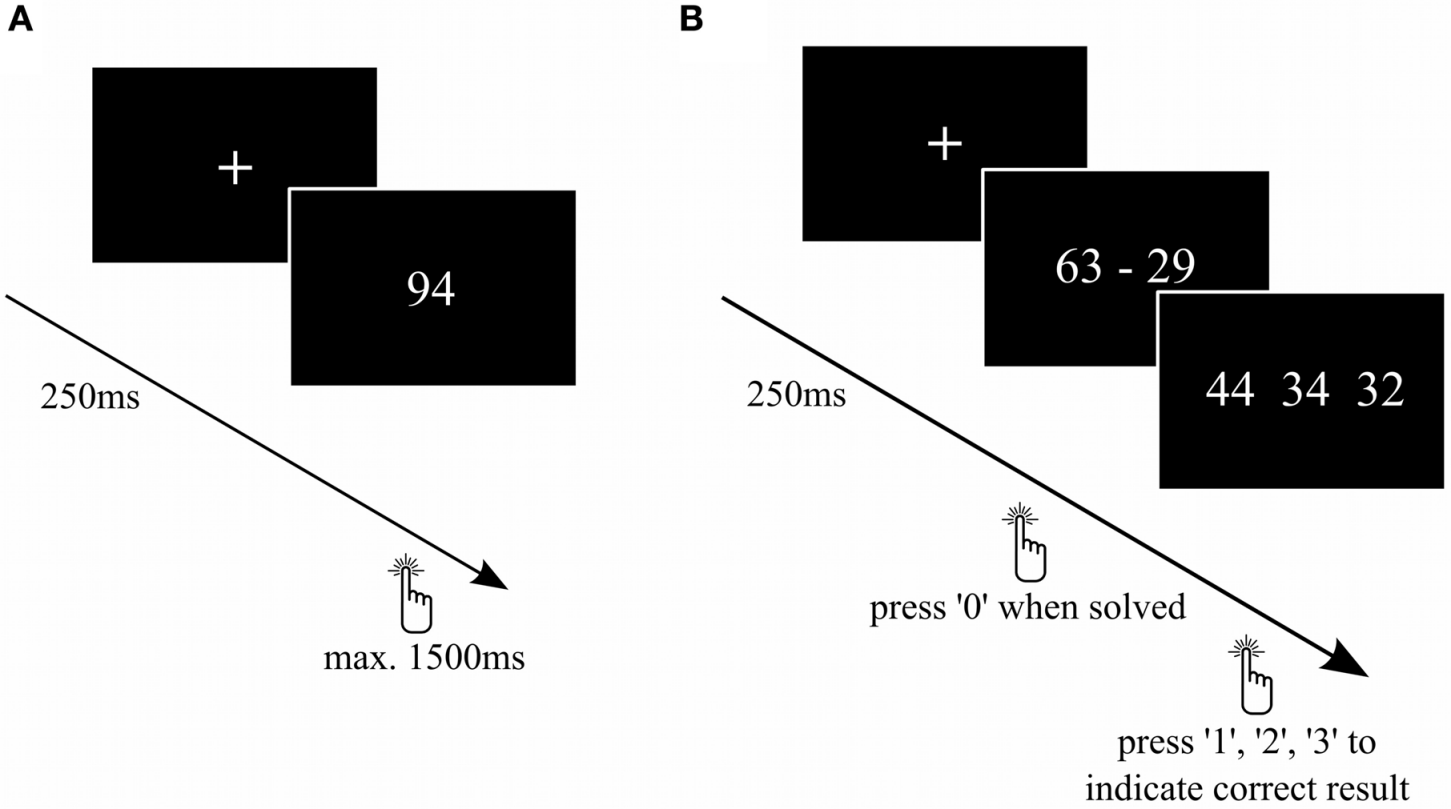
**Number comparison and complex subtraction task. (A)** On every trial of the number comparison task, participants had to decide whether a number was larger or smaller than 65. **(B)** Subjects had to calculate complex subtractions. As soon as they solved the subtraction, the participants had to press “0” and then choose the correct among the possible solutions.

**Task 1: Double-digit number comparison:** To evaluate numerical magnitude processing, we used a number comparison task (Figure 2A) similar to previous TMS studies (Göbel et al., 2001; Cappelletti et al., 2007). The subjects had to judge as fast as possible whether the presented Arabic number was bigger or smaller than 65. We used numbers from 31–99 (except 65), each presented twice. Between the stimuli, a fixation cross was presented for 250 ms.

**Task 2: Double-digit subtraction task:** To evaluate tDCS-effects on procedural mental arithmetic, a task containing 50 two-digit subtractions (Figure 2B) was presented. All subtractions were decade-overlapping and resulted in two-digit numbers. First, the subjects had to calculate the presented subtraction as fast as possible and indicate that they found the solution by pressing “0” on the number pad of the keyboard. Subsequently, three result options were displayed and the subjects had to indicate the correct solution by pressing “1”, “2”, or “3” on the number pad. Each time, 2 out of 6 distractors were randomly chosen and displayed together with the correct solution in a random order. The distractors were generated as follows: result ±1, ±2, and ±10 to prevent guessing. Between the calculations, a fixation cross was presented for 250 ms. Similar tasks have already been used in other fMRI (e.g., Ischebeck et al., 2006), but not in brain stimulation studies. The latency between stimulus presentation and first key press was analyzed as reaction time. We decided to do a two-step task rather than presenting possible solutions simultaneously with the subtraction in order to prevent participants from simply approximating the subtraction rather than computing the exact result.

#### Transcranial direct current stimulation (tDCS)

A DC-Stimulator distributed by NeuroConn GmbH (Ilmenau, Germany) was used as constant direct current source. The current was applied on the head surface using rubber electrodes, covered with saline-soaked sponges.

The active electrode was placed over P3 and/or P4 of the international EEG 10-20-system (Jasper, 1958), which corresponds with the PPC centering at the IPS (Herwig et al., 2003). The reference electrode was placed over the contralateral supraorbital region. The combination “parietal lobe—contralateral supraorbital region” was already successfully used in other tDCS-studies (e.g., Vines et al., 2006b). A current density of 0.029 mA/cm^2^ (active electrode: 35 cm^2^, 1 mA) was applied for 25 minutes, which resulted in a total charge density of 428.6 C/m^2^. The size of the reference electrode was 100 cm^2^ and therefore without influence to the underlying brain region (Nitsche et al., 2007). The current was ramped up and down for 8 s.

We used four different stimulation conditions:

**Bilateral anodal tDCS (BA):** In our setup, which we will refer to as bilateral tDCS, we attached two active electrodes over P3 and P4, respectively. The two reference electrodes were placed at the contralateral supraorbital areas.

With bilateral tDCS, we assume similar mechanisms to take effect as in traditional tDCS (one active, one reference electrode). This assumption is based on simulation studies showing that such a setup has approximately similar effects as traditional tDCS with respect to its focality and the current density distribution (Miranda et al., 2006). However, as in traditional tDCS-setups, the precise location of the maximum current density is not completely clear. Recent advances in modeling suggest that tDCS effects also depend on the tissue properties and individual anatomical differences (Salvador et al., 2010; Dmochowski et al., 2011; Neuling et al., 2012). We applied the bilateral anodal setup to maximize potential beneficial effects of tDCS given the assumption that both PPC are essential in mental arithmetic and in numerical magnitude processing

**Left-hemispheric anodal tDCS (LA):** We used the same setup as in BA. In contrast to BA, only the left tDCS-device (active electrode at P3, reference over right eyebrow) was turned on. The second tDCS-device was placed without applying any current, in order to blind the subjects with respect to the applied stimulation protocol.

**Bilateral cathodal tDCS (BC):** The electrode configuration was the same as in BA. We applied cathodal stimulation on P3 and P4 to inhibit both PPC in order to replicate previous TMS-induced impairments in numerical magnitude processing (Andres et al., 2005).

**Sham stimulation (S):** Sham stimulation was applied using the BA setup. In contrast to the active stimulation sessions, we applied current for only 30 s. This placebo-condition is known to be indistinguishable by the participants (Gandiga et al., 2006).

#### Statistical analysis

The statistical analysis was conducted using SPSS 19 (IBM Corp., Armonk, USA). As dependent variable, we used the stimulation induced changes in accuracy and response latencies (post–pre). Therewith, we controlled for offline-training effects and other constitutional biases. The data were analyzed using repeated-measures ANOVAs with the within-subject factor “stimulation” (LA, BA, BC, S). For significant effects, we computed *post-hoc* paired *t*-tests for the active stimulations vs. sham. The results were, if not indicated differently, Bonferroni corrected for multiple comparisons.

### Experiment 2

#### Participants

Sixteen healthy, right-handed subjects (9 females) aged 23.6 years (±2.4) participated in Experiment 2. For the participants in the second experiment, the same inclusion criteria as in Experiment 1 were applied (see above). The participants in both experiments did not differ in their age [*t*_(34)_ = −0.72, *p* = 0.479] or intellectual abilities [*t*_(34)_ = 0.42, *p* = 0.677] as assessed by the KAI (Kurztest für allgemeine Basisgrössen der Infomationsverarbeitung; Lehrl et al., 1992).

#### Experimental design

The subjects attended two sessions which were at least 24 h apart (Figure 1B; time between the sessions: 4.4 d ± 2.9). The protocol was the same as in the first experiment with exception of the stimulation: the subjects were stimulated with right anodal (RA) or sham (S) stimulation. The tasks as well as the stimulation duration/intensity were similar as in Experiment 1. For the stimulation, we used only one standard tDCS-device.

**Right-hemispheric anodal tDCS (RA):** The anodal electrode was placed at P4 of the 10–20 system and the reference electrode was attached to the left supraorbital region.

**Sham stimulation (S):** For sham stimulation, we used a similar setup as in RA, with the same sham stimulation parameters as in Experiment 1.

#### Statistical analysis

The statistical analysis was conducted using SPSS 19 (IBM Corp., Armonk, USA). To evaluate the tDCS-induced effects on performance, we compared the changes (post–pre) in accuracy and response latencies between the active (RA) and sham stimulation (S) using paired *t*-tests.

## Results

### Experiment 1

#### Number comparison

The analysis of the reaction times (Table 1) did not reveal any significant stimulation effects [*F*_(3, 57)_ = 1.41, *p* = 0.25].

**Table 1:** **Effects of tDCS on accuracy and reaction times in Experiment 1**.

	**S_pre_**	**S_post_**	**LA_pre_**	**LA_post_**	**BA_pre_**	**BA_post_**	**BC_pre_**	**BC_post_**
**NUMBER COMPARISON**								
Accuracy (%)	97.2 (±2.2)	96.0 (±3.1)	96.4 (±3.1)	97.1^**^ (±3.5)	97.2 (±2.3)	96.7 (±2.6)	97.2 (±2.4)	97.6 (±2.2)
Reaction time (ms)	555 (±54)	553 (±45)	569 (±56)	542 (±54)	568 (±76)	540 (±59)	565 (±65)	560 (±76)
**SUBTRACTION**								
Accuracy (%)	94.4 (±2.9)	94.2 (±3.9)	95.0 (±3.1)	95.7 (±2.8)	95.5 (±3.4)	94.7 (±3.0)	95.7 (±2.8)	94.8 (±3.6)
Reaction time (ms)	3893 (±1537)	3749 (±1642)	4262 (±2147)	3668^*^ (±1602)	3909 (±1656)	3636 (±1310)	3811 (±1596)	3575 (±1436)

Mean reaction times (±SD) and accuracies are displayed for every stimulation condition before and after tDCS for the number comparison and subtraction task. Significant improvement of the active stimulation conditions compared to sham are indicated by

** p < 0.01,

* *p < 0.05*.

**Figure 3:**
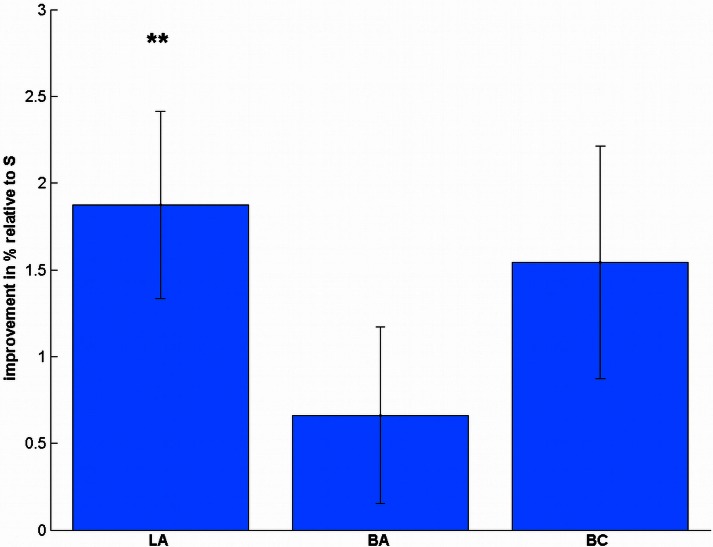
**Stimulation-induced accuracy changes in the number comparison task.** Subjects significantly improved their accuracy after LA stimulation, but not after BA or BC compared to S. ^**^p < 0.01, multiple comparison corrected.

Regarding the accuracy (Table 1), we found a significant difference between the stimulation conditions [*F*_(3, 57)_ = 4.32, *p* = 0.008]. *Post-hoc t*-tests revealed a significant effect in LA [*t*_(19) = 3.47_, *p* = 0.009, Figure 3], but not in BA [*t*_(19)_ = 1.30, *p* = 0.627] or BC [*t*_(19)_ = 2.30, *p* = 0.099] compared to S. To validate that these differences are not due to differences in the pre-test, we additionally compared the accuracies before the stimulation, where we did not find any significant difference [*F*_(3, 57)_ = 1.08, *p* = 0.364].

**Figure 4:**
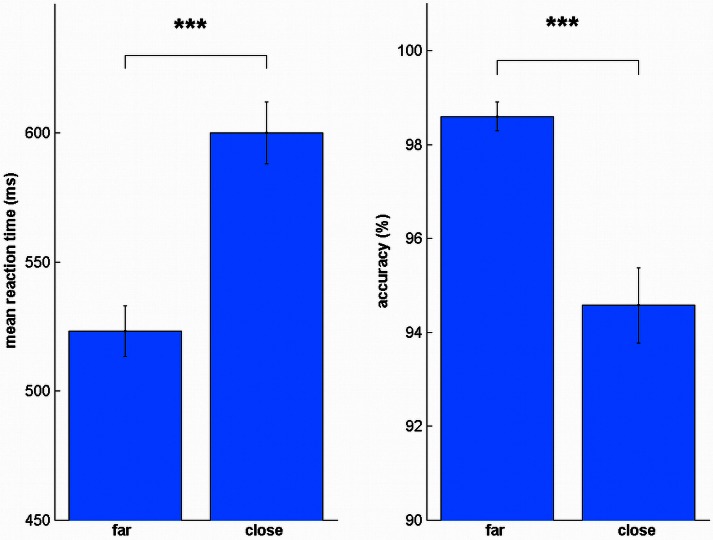
**Distance effect for reaction time and accuracy.** In all sessions, close numbers were solved significantly slower and less accurate. ^***^p < 0.001.

To evaluate the reliability of our data, we also tested whether we can replicate the distance effect (Moyer and Landauer, 1967). We split the trials into far (31–50, 80–99) and close (51–79), similar as in Cappelletti et al. (2007). We found the distance effect in all sessions for reaction times [S_pre_: *t*_(19)_ = 8.78, *p* < 0.001, all uncorrected, Figure 4; S_post_: *t*_(19)_ = 8.79, *p* < 0.001; LA_pre_: *t*_(19)_ = 6.94, *p* < 0.001; LA_post_: *t*_(19)_ = 9.91, *p* < 0.001; BA_pre_: *t*_(19)_ = 8.72, *p* < 0.001; BA_post_: *t*_(19)_ = 7.57, *p* < 0.001; BC_pre_: *t*_(19)_ = 13.32, *p* < 0.001; BC_post_: *t*_(19)_ = 4.82, *p* < 0.001] and accuracy [S_pre_: *t*_(19)_ = 6.02, *p* < 0.001, all uncorrected; S_post_: *t*_(19)_ = 3.97, *p* = 0.001; LA_pre_: *t*_(19)_ = 6.44, *p* < 0.001; LA_post_: *t*_(19)_ = 4.77, *p* < 0.001; BA_pre_: *t*_(19)_ = 3.75, *p* = 0.001; BA_post_: *t*_(19)_ = 5.75, *p* < 0.001; BC_pre_: *t*_(19)_ = 4.80, p < 0.001; BC_post_: *t*_(19)_ = 3.32, *p* = 0.004].

We also tested whether the distance effect was influenced by the stimulation condition. A repeated measures ANOVA of the differences between close and far trials between pre and post sessions [e.g., S = (S_far_post_ – S_close_post_) – (S_far_pre_ – S_close_pre_)] did not reveal any significant stimulation effect in reaction time [*F*_(3, 57)_ = 0.46, *p* = 0.714] or accuracy [*F*_(3, 57)_ = 2.55, *p* = 0.065].

#### Subtraction

**Figure 5:**
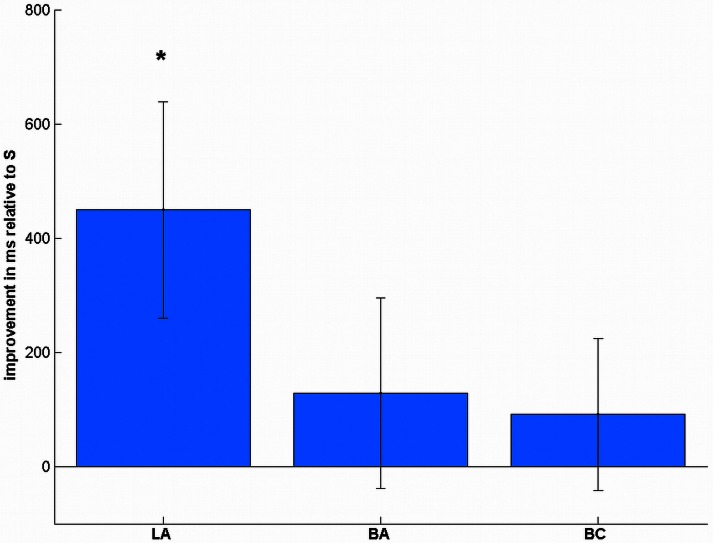
**Stimulation-induced improvements in reaction times in complex subtractions.** LA stimulation led to significant performance increases, whereas the other conditions did not change significantly. ^*^p < 0.05, multiple comparison corrected.

In the reaction times (Table 1), we found a significant effect of stimulation [*F*_(3, 57)_ = 3.01, *p* = 0.037, Figure 5]. *Post-hoc t*-tests showed a significant improvement after LA [*t*_(19)_ = 2.38, *p* = 0.048, one-tailed], but not after BA [*t*_(19)_ = 0.77, *p* = 1.00] or BC [*t*_(19)_ = 0.69, *p* = 1.00] compared to S. To ensure that these differences are not due to any differences in the pre-test, we additionally compared the mean reaction times before the stimulation, where we did not find any significant difference [*F*_(3, 57)_ = 1.43, *p* = 0.243].

All subjects solved the subtractions very accurately (Table 1). The stimulations had no influence on the accuracy [*F*_(3, 57)_ = 0.57, *p* = 0.634].

### Experiment 2

#### Number comparison

The analysis of the mean reaction times (Table 2) did not reveal any difference between the stimulations [*t*_(15)_ = 0.95, *p* = 0.359]. The accuracy (Table 2) did also not differ between the stimulation conditions [*t*_(15)_ = −0.58, *p* = 0.573].

**Table 2:** **Effects of tDCS on accuracy and reaction times in Experiment 2**.

	**S_pre_**	**S_post_**	**RA_pre_**	**RA_post_**
**NUMBER COMPARISON**				
Accuracy (%)	96.7 (±2.4)	96.6 (±2.4)	96.2 (±3.2)	96.6 (±3.4)
Reaction time (ms)	599 (±71)	576 (±63)	601 (±70)	566 (±51)
**SUBTRACTION**				
Accuracy (%)	92.9 (±4.1)	95.8 (±3.5)	91.9 (±7.6)	94.9 (±4.3)
Reaction time (ms)	4738 (±2317)	4141 (±1729)	4026 (±1128)	3690 (±875)

Mean reaction times (±SD) and accuracies are displayed for sham and RA condition before and after tDCS for the number comparison and subtraction task.

Also in this experiment, we found the distance effect for all conditions in reaction time [S_pre_: *t*_(15)_ = 5.56, *p* < 0.001, all uncorrected; S_post_: *t*_(15)_ = 4.48, *p* < 0.001; RA_pre_: *t*_(15)_ = 5.24, *p* < 0.001; RA_post_: *t*_(15)_ = 8.66, *p* < 0.001] and accuracy [S_pre_: *t*_(15)_ = 3.77, *p* = 0.002, all uncorrected; S_post_: *t*_(15)_ = 5.65, *p* < 0.001; RA_pre_: *t*_(15)_ = 4.39, *p* = 0.001; RA_post_: *t*_(15)_ = 4.36, *p* = 0.001]. There was no stimulation induced change in the distance effect for reaction times [*t*_(15)_ = 0.95, *p* = 0.359] or accuracy [*t*_(15)_ = 0.29, *p* = 0.776].

#### Subtraction

The reaction times did not change significantly due to the stimulation [*t*_(15)_ = 1.13, *p* = 0.275]. Also the accuracy did not differ significantly [*t*_(15)_ = 0.07, *p* = 0.944].

## Discussion

In our daily life, it is essential to be able to quickly and correctly access numerosities and perform more complex numerical manipulations, such as solving subtractions. Impairments of these functions, such as in DD, lead to huge challenges during schooling and professional career (Bynner and Parsons, 1997; Parsons and Bynner, 2005). In recent years, tDCS (Nitsche and Paulus, 2000) has been introduced as a promising tool to enhance cognitive functions (Cohen Kadosh et al., 2012). In this study, we therefore evaluated whether tDCS could be used to improve performance in number comparison and mental arithmetic. A first indication of a beneficial effect of tDCS in learning a new number-symbol system has been presented by Cohen Kadosh et al. (2010). The authors found improvements in the acquisition of new numerical symbols in a small sample of healthy adults after right-sided anodal stimulation of the PPC. However, other functions which are important for mathematics, such as numerical magnitude processing or mental arithmetic, have not yet been studied.

In the present study, we found that subjects responded significantly more accurately after left-sided anodal stimulation (LA) of the PPC compared to sham stimulation in a number comparison task. Furthermore, LA also induced performance improvements compared to sham in a complex subtraction task. These findings provide the first demonstration that tDCS might be helpful for improving mathematical performance in several domains.

Due to the unclear lateralization of mental arithmetic and numerical magnitude processing and the unclear interaction of both hemispheres, we also investigated the effects of simultaneous bilateral stimulation using two tDCS devices. Similar setups have been successfully administered in previous studies (e.g., Vines et al., 2008; Lindenberg et al., 2010). Our hypothesis that the simultaneous stimulation of both hemispheres would lead to more pronounced effects could, however, not be supported. We did not find any significant change in performance in both, the excitation (BA) as well as the inhibition (BC) condition. This suggests that the interaction between the two hemispheres might be more complex than, for instance, merely additive. For instance, it may be organized according to the interhemispheric inhibition (IHI) principle (Ferbert et al., 1992). IHI is well known from motor cortices (Vines et al., 2006a), and has already been suggested for the PPC in visual processing (Battelli et al., 2009). One could therefore speculate that the left PPC, which turned out to be crucially involved in the tested functions, is inhibited by the right PPC, which could be less important in the tested functions. A bilateral excitation would therefore annihilate the beneficial effect of LA. However, such an interaction in the domain of mathematics must be explored in more depth.

In our second experiment, we did not find any changes in performance after right-sided anodal PPC stimulation in number comparison or subtraction. Our findings therefore seem to contradict the findings by Cohen Kadosh et al. (2010). However, it should be noted that our study design was different in several aspects. First, our study investigated the effects of brain stimulation on the performance of mathematical tasks, whereas Cohen Kadosh et al. (2010) investigated the learning of number-symbol associations over a period of several days. Second, we used a different stimulation protocol. Cohen Kadosh et al. (2010) stimulated while the participants learned symbol-numerosity-associations. We used an offline-tDCS protocol where participants performed the tasks before and after the stimulation. Finally, we assessed different aspects of mathematics. Cohen Kadosh et al. (2010) were interested in the learning of symbol-numerosity-associations, whereas we investigated magnitude processing and mental arithmetic.

Our finding that only LA improves performance in numerical magnitude processing is in line with the majority of the TMS studies which found an impairment after left-sided inhibition (Göbel et al., 2001; Sandrini et al., 2004; Andres et al., 2005; Rusconi et al., 2005; Knops et al., 2006; Cappelletti et al., 2007; Dormal et al., 2008). TMS studies, which investigated right-sided inhibition, on the other hand, led to inconsistent results (Göbel et al., 2001; Sandrini et al., 2004; Andres et al., 2005; Cappelletti et al., 2007; Cohen Kadosh et al., 2007; Dormal et al., 2008).

Likewise, our finding that the left PPC is crucially involved in subtractions is in line with fMRI-studies which usually show a slightly left-lateralized activation pattern (Arsalidou and Taylor, 2011). Since no previous brain stimulation study has shown a beneficial effect of brain stimulation on subtractions, our findings crucially further the understanding of the PPC during complex subtractions.

Although we have convincing evidence that LA improves numerical magnitude processing and complex mental arithmetic, the present study has one minor limitation. Because we wanted to avoid that our results might be confounded by effects which are not related to tDCS or by individual differences, such as mathematical competence which is known to influence neural processes (Grabner et al., 2007), we decided to conduct a within-subjects design. The participants therefore attended four sessions in Experiment 1. To ensure that training effects do not have any influence on the results, we counter-balanced the stimulation conditions across sessions. Furthermore, we compared performance changes after the stimulation to the baseline performance, assessed before stimulation in every session.

The present findings encourage a further exploration of the beneficial effects of left anodal tDCS at the PPC in the domain of mathematics. Although we have shown its beneficial effect on magnitude processing and mental arithmetic, the effect of tDCS on several aspects of mathematics still needs to be evaluated. In the domain of mental arithmetic, it should be tested whether LA also has beneficial effects on operations and strategies other than the procedures we tested in this study. For example, it should be tested whether small additions, which are usually retrieved from memory, also benefit from LA. Furthermore, the effect of tDCS on the acquisition of arithmetic skills, such as the learning of arithmetic facts and strategies, should also be evaluated, because these are key competencies taught in school. Not only should the effects of LA be tested behaviorally, but also the neural mechanism underlying these effects should be studied using neuroimaging methods such as electroencephalography and fMRI. If such studies reinforce the usefulness of LA, it has to be assessed whether these beneficial effects also improve performance in people with mathematical disabilities, such as DD or acalculia, where remediation is still sparse (Cohen Kadosh and Walsh, 2007).

### Conflict of interest statement

The authors declare that the research was conducted in the absence of any commercial or financial relationships that could be construed as a potential conflict of interest.

## References

[B1] Andres M. Pelgrims B. Michaux N. Olivier E. Pesenti M. (2011). Role of distinct parietal areas in arithmetic: an fMRI-guided TMS study. Neuroimage 54, 3048–3056 10.1016/j.neuroimage.2010.11.009 21073958

[B2] Andres M. Seron X. Olivier E. (2005). Hemispheric lateralization of number comparison. Cogn. Brain Res. 25, 283–290 10.1016/j.cogbrainres.2005.06.002 16005617

[B3] Arsalidou M. Taylor M. J. (2011). Is 2 + 2 = 4? Meta-analyses of brain areas needed for numbers and calculations. Neuroimage 54, 2382–2393 10.1016/j.neuroimage.2010.10.009 20946958

[B4] Ansari D. (2007). Does the parietal cortex distinguish between “10,” “ten,” and ten dots? Neuron 53, 165–167 10.1016/j.neuron.2007.01.001 17224400

[B5] Ansari D. (2008). Effects of development and enculturation on number representation in the brain. Nat. Rev. Neurosci. 9, 278–291 10.1038/nrn2334 18334999

[B6] Battelli L. Alvarez G. A. Carlson T. Pascual-Leone A. (2009). The role of the parietal lobe in visual extinction studied with transcranial magnetic stimulation. J. Cogn. Neurosci. 21, 1946–1955 10.1162/jocn.2008.21149 18855545 PMC3366148

[B7] Beeli G. Casutt G. Baumgartner T. Jäncke L. (2008a). Modulating presence and impulsiveness by external stimulation of the brain. Behav. Brain Funct. 4, 33 10.1186/1744-9081-4-33 18680573 PMC2529286

[B8] Beeli G. Koeneke S. Gasser K. Jäncke L. (2008b). Brain stimulation modulates driving behavior. Behav. Brain Funct. 4, 34 10.1186/1744-9081-4-34 18684333 PMC2527008

[B9] Bindman L. J. Lippold O. C. Refearn J. W. (1964). The action of brief polarizin currents on the cerebral cortex of the rat (1) during current flow and (2) in the production of long-lasting after-effects. J. Physiol. 172, 369–382 14199369 10.1113/jphysiol.1964.sp007425 PMC1368854

[B10] Boggio P. S. Sultani N. Fecteau S. Merabet L. Mecca T. Pascual-Leone A. (2008). Prefrontal cortex modulation using transcranial DC stimulation reduces alcohol craving: a double-blind, sham-controlled study. Drug Alcohol Depend. 92, 55–60 10.1016/j.drugalcdep.2007.06.011 17640830

[B11] Bynner J. Parsons S. (1997). Does Numeracy Matter? London: Basic Skills Agency

[B12] Cappelletti M. Barth H. Fregni F. Spelke E. S. Pascual-Leone A. (2007). rTMS over the intraparietal sulcus disrupts numerosity processing. Exp. Brain Res. 179, 631–642 10.1007/s00221-006-0820-0 17216413 PMC2567820

[B13] Cerruti C. Schlaug G. (2008). Anodal transcranial direct current stimulation of the prefrontal cortex enhances complex verbal associative thought. J. Cogn. Neurosci. 21, 1980–1987 10.1162/jocn.2008.21143 18855556 PMC3005595

[B14] Cohen Kadosh R. Cohen Kadosh K. Schuhmann T. Kaas A. Goebel R. Henik A. (2007). Virtual dyscalculia induced by parietal-lobe TMS impairs automatic magnitude processing. Curr. Biol. 17, 689–693 10.1016/j.cub.2007.02.056 17379521

[B15] Cohen Kadosh R. Levy N. O'Shea J. Shea N. Savulescu J. (2012). The neuroethics of non-invasive brain stimulation. Curr. Biol. 22, R108–R111 10.1016/j.cub.2012.01.013 22361141 PMC4347660

[B16] Cohen Kadosh R. Soskic S. Iuculano T. Kanai R. Walsh V. (2010). Modulating neuronal activity produces specific and long-lasting changes in numerical competence. Curr. Biol. 20, 2016–2020 10.1016/j.cub.2010.10.007 21055945 PMC2990865

[B17] Cohen Kadosh R. Walsh V. (2007). Dyscalculia. Curr. Biol. 17, R946–R947 10.1016/j.cub.2007.08.038 18029243

[B18] Croarkin P. E. Wall C. A. Lee J. (2011). Applications of transcranial magnetic stimulation (TMS) in child and adolescent psychiatry. Int. Rev. Psychiatry 23, 445–453 10.3109/09540261.2011.623688 22200134

[B19] Dehaene S. Piazza M. Pinel P. Cohen L. G. (2003). Three parietal circuits for number processing. Cogn. Neuropsychol. 20, 487–506 10.1080/02643290244000239 20957581

[B20] Dmochowski J. P. Datta A. Bikson M. Su Y. Parra L. C. (2011). Optimized multi-electrode stimulation increases focality and intensity at target. J. Neural Eng. 8, 1–16 10.1088/1741-2560/8/4/046011 21659696

[B21] Dormal V. Andres M. Pesenti M. (2008). Dissociation of numerosity and duration processing in the left intraparietal sulcus: a transcranial magnetic stimulation study. Cortex 44, 462–469 10.1016/j.cortex.2007.08.011 18387579

[B22] Ferbert A. Priori A. Rothwell J. C. Day B. L. Colebatch J. G. Marsden C. D. (1992). Interhemispheric inhibition of the human motor cortex. J. Physiol. 453, 525–546 1464843 10.1113/jphysiol.1992.sp019243 PMC1175572

[B23] Fias W. Lammertyn J. Reynvoet B. Dupont P. Orban G. A. (2003). Parietal representation of symbolic and nonsymbolic magnitude. J. Cogn. Neurosci. 15, 47–56 10.1162/089892903321107819 12590842

[B24] Fregni F. Boggio P. S. Nitsche M. A. Bermpohl F. Antal A. Feredoes E. (2005). Anodal transcranial direct current stimulation of prefrontal cortex enhances working memory. Exp. Brain Res. 166, 23–30 10.1007/s00221-005-2334-6 15999258

[B25] Gandiga P. C. Hummel F. C. Cohen L. G. (2006). Transcranial DC stimulation (tDCS): a tool for double-blind sham-controlled clinical studies in brain stimulation. Clin. Neurophysiol. 117, 845–850 10.1016/j.clinph.2005.12.003 16427357

[B26] Geary D. C. (1993). Mathematical disabilities: cognitive, neuropsychological, and genetic components. Psychol. Bull. 114, 345–362 10.1037/0033-2909.114.2.345 8416036

[B27] Geary D. C. (2010). Mathematical disabilities: reflections on cognitive, neuropsychological, and genetic components. Learn. Individ. Differ. 20, 130–130 10.1016/j.lindif.2009.10.008 20161681 PMC2821095

[B28] Göbel S. M. Rushworth M. F. Walsh V. (2006). Inferior parietal rtms affects performance in an addition task. Cortex 42, 774–781 10.1016/S0010-9452(08)70416-7 16909638

[B29] Göbel S. M. Walsh V. Rushworth M. F. (2001). The mental number line and the human angular gyrus. Neuroimage 14, 1278–1289 10.1006/nimg.2001.0927 11707084

[B30] Grabner R. H. Ansari D. Koschutnig K. Reishofer G. Ebner F. Neuper C. (2009). To retrieve or to calculate? Left angular gyrus mediates the retrieval of arithmetic facts during problem solving. Neuropsychologia 47, 604–608 10.1016/j.neuropsychologia.2008.10.013 19007800

[B31] Grabner R. H. Ansari D. Reishofer G. Stern E. Ebner F. Neuper C. (2007). Individual differences in mathematical competence predict parietal brain activation during mental calculation. Neuroimage 38, 346–356 10.1016/j.neuroimage.2007.07.041 17851092

[B32] Hagner M. (2004). Geniale Gehirne - Zur Geschichte der Elitegehirnforschung. Göttingen: Wallstein Verlag

[B33] Herwig U. Satrapi P. Schönfeldt-Lecuona C. (2003). Using the international 10-20 EEG system for positioning of transcranial magnetic stimulation. Brain Topogr. 16, 95–99 10.1023/B:BRAT.0000006333.93597.9d 14977202

[B34] Holtzheimer P. E. 3rd Kosel M. Schlaepfer T. (2012). Brain stimulation therapies for neuropsychiatric disease. Handb. Clin. Neurol. 106, 681–695 10.1016/B978-0-444-52002-9.00041-3 22608652

[B35] Hummel F. C. Cohen L. G. (2006). Non-invasive brain stimulation: a new strategy to improve neurorehabilitation after stroke? Lancet Neurol. 5, 708–712 10.1016/S1474-4422(06)70525-7 16857577

[B36] Im C.-H. Jung H.-H. Choi J.-D. Lee S. Y. Jung K.-Y. (2008). Determination of optimal electrode positions for transcranial direct current stimulation (tDCS). Phys. Med. Biol. 53, N219–N225 10.1088/0031-9155/53/11/N03 18490807

[B37] Ischebeck A. Zamarian L. Siedentopf C. Koppelstätter F. Benke T. Felber S. (2006). How specifically do we learn? Imaging the learning of multiplication and subtraction. Neuroimage 30, 1365–1375 10.1016/j.neuroimage.2005.11.016 16413795

[B38] Jasper H. H. (1958). The ten-twenty electrode system of the international federation. Electroencephalogr. Clin. Neurophysiol. 10, 370–375 10590970

[B39] Knops A. Nuerk H.-C. Sparing R. Foltys H. Willmes K. (2006). On the functional role of human parietal cortex in number processing: how gender mediates the impact of a “virtual lesion” induced by rTMS. Neuropsychologia 44, 2270–2283 10.1016/j.neuropsychologia.2006.05.011 16828812

[B40] Landerl K. Bevan A. Butterworth B. (2004). Developmental dyscalculia and basic numerical capacities: a study of 8–9-year-old students. Cognition 93, 99–125 10.1016/j.cognition.2003.11.004 15147931

[B41] Lehrl S. Gallwitz A. Blaha L. (1992). Kurztest Für Allgemeine Basisgrössen der Informationsverarbeitung, K. A. I., 3rd Edn Ebersberg: VLESS Verlag

[B42] Lindenberg R. Renga V. Zhu L. L. Nair D. Schlaug G. (2010). Bihemispheric brain stimulation facilitates motor recovery in chronic stroke patients. Neurology 75, 2176–2184 10.1212/WNL.0b013e318202013a 21068427 PMC3013585

[B43] Miranda P. C. Lomarev M. Hallett M. (2006). Modeling the current distribution during transcranial direct current stimulation. Clin. Neurophysiol. 117, 1623–1629 10.1016/j.clinph.2006.04.009 16762592

[B44] Monti A. Cogiamanian F. Marceglia S. Ferrucci R. Mameli F. Mrakic-Sposta S. (2008). Improved naming after transcranial direct current stimulation in aphasia. J. Neurol. Neurosurg. Psychiatr. 79, 451–453 10.1136/jnnp.2007.135277 18096677

[B45] Moyer R. S. Landauer T. K. (1967). Time required for judgements of numerical inequality. Nature 215, 1519–1520 10.1038/2151519a0 6052760

[B46] Neuling T. Wagner S. Wolters C. H. Zaehle T. Herrmann C. S. (2012). Finite-element model predicts current density distribution for clinical applications of tDCS and tACS. Front. Psychiatry 3:83 10.3389/fpsyt.2012.00083 23015792 PMC3449241

[B47] Nieder A. (2005). Counting on neurons: the neurobiology of numerical competence. Nat. Rev. Neurosci. 6, 177–190 10.1038/nrn1626 15711599

[B48] Nieder A. Dehaene S. (2009). Representation of number in the brain. Annu. Rev. Neurosci. 32, 185–208 10.1146/annurev.neuro.051508.135550 19400715

[B49] Nitsche M. A. Boggio P. S. Fregni F. Pascual-Leone A. (2009). Treatment of depression with transcranial direct current stimulation (tDCS): a review. Exp. Neurol. 219, 14–19 10.1016/j.expneurol.2009.03.038 19348793

[B50] Nitsche M. A. Doemkes S. Karaköse T. Antal A. Liebetanz D. Lang N. (2007). Shaping the effects of transcranial direct current stimulation of the human motor cortex. J. Neurophysiol. 97, 3109–3117 10.1152/jn.01312.2006 17251360

[B51] Nitsche M. A. Paulus W. (2000). Excitability changes induced in the human motor cortex by weak transcranial direct current stimulation. J. Physiol. 527(Pt 3), 633–639 10.1111/j.1469-7793.2000.t01-1-00633.x 10990547 PMC2270099

[B52] Parsons S. Bynner J. (2005). Does Numeracy Matter More? London: National Research and Development Centre for adult literacy and numeracy

[B53] Peng Z. Chen X.-Q. Gong S.-S. (2012). Effectiveness of repetitive transcranial magnetic stimulation for chronic tinnitus: a systematic review. Otolaryngol. Head Neck Surg. 147, 817–825 10.1177/0194599812458771 22941756

[B54] Pinel P. Dehaene S. Rivière D. LeBihan D. (2001). Modulation of parietal activation by semantic distance in a number comparison task. Neuroimage 14, 1013–1026 10.1006/nimg.2001.0913 11697933

[B55] Rusconi E. Walsh V. Butterworth B. (2005). Dexterity with numbers: rTMS over left angular gyrus disrupts finger gnosis and number processing. Neuropsychologia 43, 1609–1624 10.1016/j.neuropsychologia.2005.01.009 16009243

[B56] Salvador R. Mekonnen A. Ruffini G. Miranda P. C. (2010). Modeling the electric field induced in a high resolution realistic head model during transcranial current stimulation. Conf. Proc. IEEE Eng. Med. Biol. Soc. 2010, 2073–2076 10.1109/IEMBS.2010.5626315 21095946

[B57] Sandrini M. Rossini P. M. Miniussi C. (2004). The differential involvement of inferior parietal lobule in number comparison: a rTMS study. Neuropsychologia 42, 1902–1909 10.1016/j.neuropsychologia.2004.05.005 15381020

[B58] Sandrini M. Rusconi E. (2009). A brain for numbers. Cortex 45, 796–803 10.1016/j.cortex.2008.09.002 18976990

[B59] Schlaug G. Renga V. Nair D. (2008). Transcranial direct current stimulation in stroke recovery. Arch. Neurol. 65, 1571–1576 10.1001/archneur.65.12.1571 19064743 PMC2779259

[B60] Seymour S. E. Reuter-Lorenz P. A. Gazzaniga M. S. (1994). The disconnection syndrome. Basic findings reaffirmed. Brain 117(Pt 1), 105–115 10.1093/brain/117.1.105 8149205

[B61] Vines B. W. Cerruti C. Schlaug G. (2008). Dual-hemisphere tDCS facilitates greater improvements for healthy subjects' non-dominant hand compared to uni-hemisphere stimulation. BMC Neurosci. 9:103 10.1186/1471-2202-9-103 18957075 PMC2584652

[B62] Vines B. W. Nair D. Schlaug G. (2006a). Contralateral and ipsilateral motor effects after transcranial direct current stimulation. Neuroreport 17, 671–674 16603933 10.1097/00001756-200604240-00023

[B63] Vines B. W. Schnider N. M. Schlaug G. (2006b). Testing for causality with transcranial direct current stimulation: pitch memory and the left supramarginal gyrus. Neuroreport 17, 1047–1050 10.1097/01.wnr.0000223396.05070.a2 16791101 PMC3005564

